# The Human Brain

**Published:** 1844-11

**Authors:** 


*The Human Brain.*—The weight of this organ in adult white males, varies from 3 lbs. 2 oz. to 4 lbs. 6 oz. Troy weight. The brain of men distinguished for their intellectual power is usually large. That of Cuvier was one-third larger than an ordinary brain. It weighed 4 lbs. 11 oz. 4 dr. and 30 grains. The brain of idiots is usually small. That of an idiot 50 years old, weighed but 1 lb. 8 oz. and 4 dr., and that of anather aged 40, 1 lb. 11 oz. 4 dr.

The male brain is on the average heavier than the female, yet in proportion to the weight of the whole body, it is rather less heavy. The intellect is not absolutely proportioned to the size of the brain, but it is proportioned to the size of the hemispheres, and especially to the extent of their surfaces. In emaciation this organ does not diminish in proportion as the rest of the body does. Hence in many diseases accompanied with great emaciation of body the mind remains active and vigorous.—*Jour. of Insanity.*

